# The effects of graded levels of calorie restriction: VI. Impact of short-term graded calorie restriction on transcriptomic responses of the hypothalamic hunger and circadian signaling pathways

**DOI:** 10.18632/aging.100895

**Published:** 2016-02-23

**Authors:** Davina Derous, Sharon E. Mitchell, Cara L. Green, Luonan Chen, Jing‐Dong J. Han, Yingchun Wang, Daniel E.L. Promislow, David Lusseau, John R. Speakman, Alex Douglas

**Affiliations:** ^1^ Institute of Biological and Environmental Sciences, University of Aberdeen, Aberdeen, Scotland, AB24 2TZ, UK; ^2^ Centre for Genome Enabled Biology and Medicine, University of Aberdeen, Aberdeen, Scotland, AB24 3RL, UK; ^3^ Key laboratory of Systems Biology, Innovation Center for Cell Signaling Network, Institute of Biochemistry and Cell Biology, Shanghai Institute of Biological Sciences, Chinese Academy of Sciences, Shanghai, 200031, China; ^4^ Chinese Academy of Sciences Key Laboratory of Computational Biology, Chinese Academy of Sciences‐Max Planck Partner Institute for Computational Biology, Shanghai Institutes for Biological Sciences, Chinese Academy of Sciences, Shanghai, 200031, China; ^5^ State Key laboratory of Molecular Developmental Biology, Institute of Genetics and Developmental Biology, Chinese Academy of Sciences, Chaoyang, Beijing, 100101, China; ^6^ Department of Pathology and Department of Biology, University of Washington at Seattle, Seattle, WA 98195, USA

**Keywords:** calorie restriction, circadian rhythm, hunger, hypothalamus, transcriptomics

## Abstract

Food intake and circadian rhythms are regulated by hypothalamic neuropeptides and circulating hormones, which could mediate the anti‐ageing effect of calorie restriction (CR). We tested whether these two signaling pathways mediate CR by quantifying hypothalamic transcripts of male C57BL/6 mice exposed to graded levels of CR (10 % to 40 %) for 3 months. We found that the graded CR manipulation resulted in upregulation of core circadian rhythm genes, which correlated negatively with circulating levels of leptin, insulin‐like growth factor 1 (IGF‐1), insulin, and tumor necrosis factor alpha (TNF‐α). In addition, key components in the hunger signaling pathway were expressed in a manner reflecting elevated hunger at greater levels of restriction, and which also correlated negatively with circulating levels of insulin, TNF‐α, leptin and IGF‐1. Lastly, phenotypes, such as food anticipatory activity and body temperature, were associated with expression levels of both hunger genes and core clock genes. Our results suggest modulation of the hunger and circadian signaling pathways in response to altered levels of circulating hormones, that are themselves downstream of morphological changes resulting from CR treatment, may be important elements in the response to CR, driving some of the key phenotypic outcomes.

## INTRODUCTION

Calorie restriction (CR) is a well-established noninvasive method that reduces the rate of ageing, increases lifespan and delays the onset of age-associated diseases in a wide range of taxa [1–6]. However, the underlying mechanisms by which CR exerts its antiageing effects are still unclear [7]. The response of the brain to CR includes at least three different coordinated processes: (1) development of hunger and co-ordination of food seeking behaviors and circadian rhythms; (2) co-ordination of responses that mobilize body fuel stores and reduce energy expenditure, including physiological and behavioral responses; and (3) an increase in resistance to brain damage and pathology and co-ordination of cellular resistance in the periphery (reviewed in [8]). Hunger signaling and circadian rhythms are both regulated by the hypothalamus and in this paper we explored the responses of these processes to CR.

Food intake is regulated by many neuropeptides and signaling molecules in the hypothalamus which form a complex interacting network. Research over the past two decades, since the discovery of leptin [9], has started to unravel this complex signaling system and identified several key signaling molecules that affect food intake [10,11], with discoveries still emerging [12,13]. Four important neuropeptides involved in hunger signaling are neuropeptide Y (NPY), agoutirelated peptide (AgRP), pro-opiomelanocortin (POMC) and cocaine- and amphetamine-regulated transcript (CART). Increased levels of NPY and AgRP both stimulate food intake, while elevated levels of POMC and CART reduce intake. AgRP/NPY and POMC/CART reside on different neuronal populations in the arcuate nucleus of the hypothalamus [14]. Recent work suggests that AgRP is also intimately involved in hunger related activity patterns [12,13]. Both AgRP/NPY and POMC/CART neuronal types have multiple receptor populations, including both insulin and leptin receptors, which enables them to respond to the nutritional status of the individual. Specifically leptin and insulin stimulates POMC and CART and inhibit AgRP and NPY [15]. Changes in peripheral levels of these hormones have previously been implicated in mediating the effects of CR [16,17].

Previous work in mice exposed to short-term (100 days) CR show altered expression of these key elements of the hunger signaling pathway, including elevated levels of NPY and AgRP and reduced levels of POMC and CART, when compared to *ad libitum* fed controls [18]. Upon re-feeding, after a period of CR, the hyperphagic response suggests that hunger remained even after energy balance was re-established [18]. This elevated ‘hunger profile’ might be a major factor contributing to the beneficial effects of CR [19]. If this is the case, we would anticipate that, based on the linear relationship between the percentage of CR and the increase in lifespan (reviewed in [6]), a graded increase in the level of CR would lead to graded levels of expression in these four key genes and graded expression of both up and downstream connected components of the intracellular signaling cascades. We therefore also expected genes involved in the leptin, insulin, and other hormonal signaling pathways to be altered in a graded manner and this would be related to the expression of the four key hunger genes (NPY, AgRP, POMC and CART). Furthermore, mice exposed to CR showed specific behavioral changes, such as elevated food anticipatory activity (FAA) within a short time before feeding [20,21], and this FAA is probably regulated by AgRP and POMC [12]. These responses also include a drop in body temperature and the emergence of torpor at 30 % and 40 % CR [22,23].

Food seeking behavior and temperature regulation are under circadian control [24], which is established centrally by the suprachiasmatic nucleus (SCN) located in the hypothalamus [25]. Circadian regulation is linked to metabolic homeostasis, and dysregulation can lead to metabolic diseases [26]. The interaction between circadian rhythms and metabolism is complicated, and many signals contribute to this regulation. Food intake has been found to influence circadian rhythms [26], and SCN cells have insulin, leptin and other hormone receptors [27,28]. Insulin in particular is thought to play an important role as a signaling hormone linking metabolism and circadian rhythms [29,30]. Furthermore circadian rhythms are influenced by age [31]. Older mice lose the ability to synchronize as well with the environment, although the circadian rhythms remain [32]. Desynchronization of circadian rhythms has a negative effect on longevity [33] and CR synchronizes these rhythms in the SCN [24]. This suggests CR might protect against age-associated loss of circadian rhythm synchronization [34]. Therefore, we expected that the response to CR would be to alter the expression of core clock genes, with circulating hormones playing a central role in this modulation.

We hypothesize that peripheral hormone-driven changes in gene expression in the hunger and circadian signaling pathways in the hypothalamus may be fundamental elements of the response to CR, and that these changes could mediate some of the beneficial impacts of CR. We tested these predictions using the hypothalamic transcriptome of mice exposed to different levels of CR. The phenotypic responses of these same mice (body composition, food intake, hormone levels, body temperature, use of torpor and physical activity behavior) have been extensively characterized [22,23,35,36].

## RESULTS

### Graded CR responses involve biological processes related to circadian rhythms and hunger

**Figure 1:**
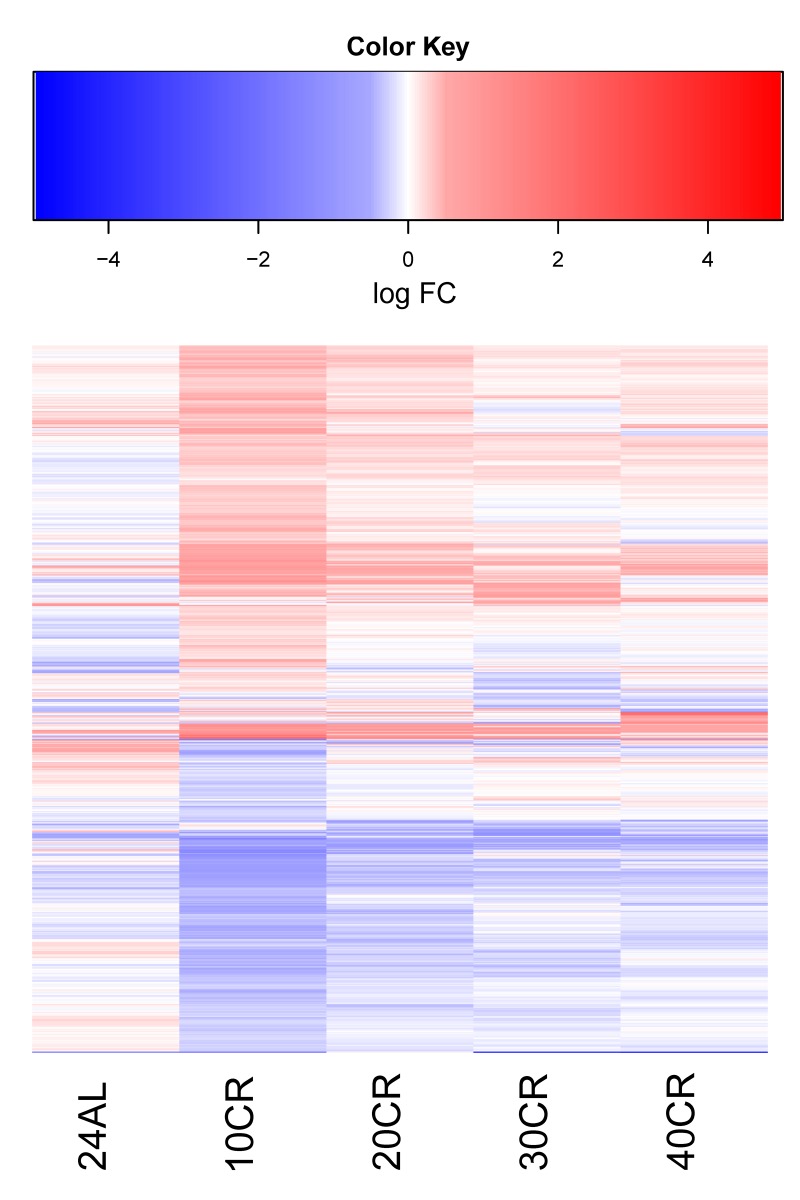
Log Fold change of differentially expressed genes in at least one treatment relative to 12 hour *ad libitum* feeding (12AL). Blue indicates down-regulation and red upregulation relative to 12AL. 10CR, 20CR, 30CR and 40CR refer to 10 %, 20 %, 30 % and 40 % restriction and 24AL to 24h *ad libitum* feeding.

In total, 1086 genes of the 14013 genes were identified to have a significantly altered expression in at least one of the CR levels (i.e. 24 hours *ad libitum* (24AL), 10CR, 20CR, 30CR, 40CR) relative to 12 hours *ad libitum* (12AL) (Likelihood ratio test p-value < 0.05). The expression profile of these 1086 genes relative to 12AL (log2 fold changes (log FC)) had a similar expression at 10CR, 20CR, 30CR and 40CR (Fig 1).

**Figure 2:**
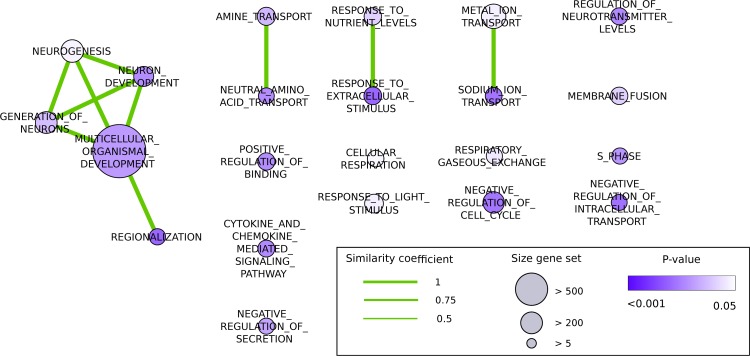
Significantly differentially regulated biological processes after three months of CR treatment, based on genes altered across CR, visualized as an Enrichment map (Cytoscape). The nodes represent biological processes, and edges represent overlap between genes in these processes. The color of the nodes represents the significance according to the p-value (white: p-value = 0.05, purple: p-value < 0.01). The size of the nodes corresponds to the size of the gene set. The width of edges is based on similarity coefficients (> 0.5) between the nodes, derived from the overlap of the gene sets underlying the processes.

Gene Set Enrichment Analysis (GSEA) revealed that these genes were significantly (p-value < 0.05) enriched in 22 biological processes (Fig 2). These processes were related to transport (e.g. ‘amine transport’, ‘neutral amino acid transport’, ‘metal ion transport’), regulation of neurotransmitter levels, cytokine and chemokine mediated signaling pathways, circadian rhythms (‘response to light stimulus’), hunger (‘response to nutrient levels’), metabolism of neurons (e.g. ‘neurogenesis’, ‘neuron development’ and ‘generation of neurons’) and the cell cycle (e.g. ‘negative regulation of cell cycle’ and ‘S phase’).

Based on the Benjamini Hochberg adjusted p-value (FDR < 0.05) none of the genes were significant at 24AL and 10CR relative to 12AL, 1 gene was significant at 20CR, 2 genes at 30CR and 220 genes at 40CR. Therefore significantly differentially expressed genes (DEGs) were based on a cut off p-value < 0.05 and an absolute log2 fold change (log FC) > 0.5 to 12AL. The number of DEGs relative to 12AL increased as the level of CR increased with exception of 30CR (i.e. for 24AL, 10CR, 20CR, 30CR, 40CR there were 103, 117, 152, 133 and 385 DEGs respectively). Pathway analysis and upstream transcription factor identification for each CR level and 24AL relative to 12AL highlighted a different transcriptomic response for 24AL (Supplemental results).

### Graded CR responses involved a negative correlation between genes signaling hunger and circulating hormone levels

**Figure 3:**
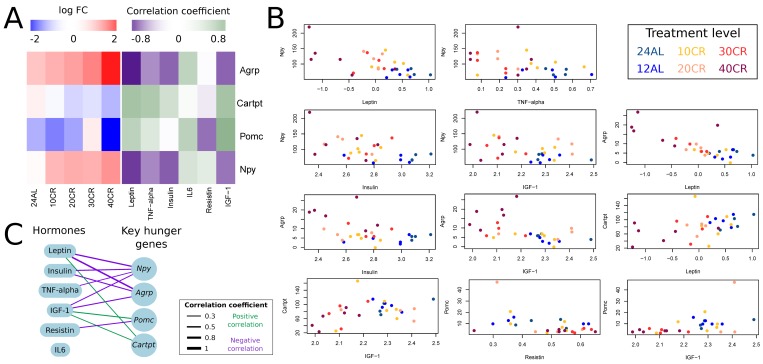
Effect of graded calorie restriction on circulating hormone levels and genes involved in hunger signaling. (**A**) Genes involved in hunger signaling based on log fold change relative to *ad libitum* feeding for 12h per day (12AL) and their correlation coefficient with circulating hormone levels. Blue indicates down-regulation and red upregulation relative to 12AL. Purple indicates a negative correlation coefficient and green a positive correlation with circulating hormone levels. 10CR, 20CR, 30CR and 40CR refers to 10 %, 20 %, 30 % and 40 % restriction and 24AL to 24h *ad libitum* feeding. (**B**) Expression levels of key hunger genes plotted against circulating hormone levels. Those genes with a significant correlation based on p-value < 0.05 are included in the plot. (**C**) Overview of key hunger signaling genes correlating with circulating hormone levels. The lines connecting genes and hormones represent correlations and the width of these lines indicates the strength of the correlation coefficient. Green indicates a positive correlation and purple a negative correlation.

We explored the response of key hunger genes (*Npy*, *Agrp*, *Cartpt* and *Pomc*) to graded CR and how these genes correlated (Pearson correlation) with circulating hormone levels (hormone data from [36]). The expression of genes *Npy* and *Agrp* increased relative to CR level; and expression of *Cartpt* and *Pomc* decreased relative to CR level (Fig 3A). *Npy* was significantly upregulated at 10CR, 20CR, 30CR and at 40CR. *Agrp* was significantly upregulated at 30CR and 40CR. *Pomc* and *Cartpt* were significantly downregulated at 40CR (Table 1). Expression levels of *Agrp* correlated negatively with leptin, insulin and IGF-1. *Cartpt* correlated positively with leptin and IGF-1. *Pomc* correlated negatively with resistin and positively with IGF-1. Lastly *Npy* correlated negatively with leptin, TNF-α, insulin and IGF-1 (Fig 3B and 3C) (Table 2).

**Table 1:** Expression levels of key hunger genes relative to 12AL

	24AL		10CR		20CR		30CR		40CR	
	logFC	p-value	logFC	p-value	logFC	p-value	logFC	p-value	logFC	p-value
*Agrp*	0.509	0.352	0.670	0.202	0.823	0.139	1.078	0.050	1.992	<0.001
*Cartpt*	0.125	0.659	−0.062	0.822	−0.367	0.225	−0.270	0.371	−0.634	0.018
*Pomc*	−0.694	0.338	−1.104	0.115	−0.789	0.303	0.169	0.822	−2.242	0.001
*Npy*	0.028	0.922	0.628	0.022	0.719	0.015	0.675	0.022	0.947	<0.001

**Table 2:** Correlations between expression levels of key hunger genes and circulating hormones

	Leptin		TNF-α		Insulin		IL6		Resistin		IGF-1	
	*r*	p-value	*r*	p-value	*r*	p-value	*r*	p-value	*r*	p-value	*r*	p-value
*Agrp*	−0.741	<0.001	−0.362	0.053	−0.494	0.006	0.271	0.155	−0.010	0.961	−0.502	0.006
*Cartpt*	0.382	0.041	0.340	0.071	0.200	0.298	0.025	0.899	−0.071	0.715	0.384	0.039
*Pomc*	0.282	0.138	0.145	0.454	−0.011	0.955	0.294	0.121	−0.461	0.012	0.474	0.009
*Npy*	−0.629	<0.001	−0.384	0.040	−0.524	0.004	0.172	0.371	0.119	0.539	−0.445	0.016

**Figure 4:**
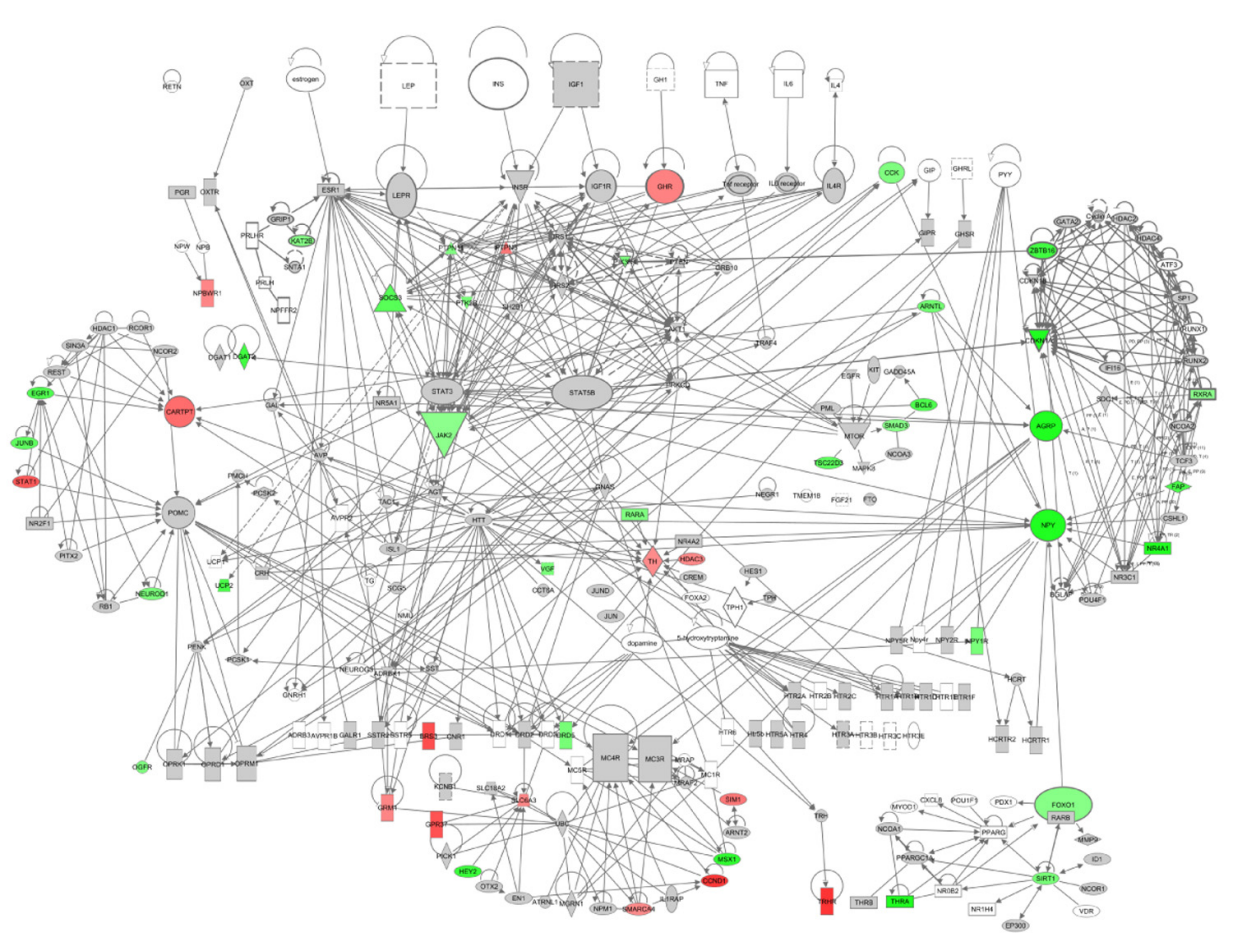
The hunger signaling pathway constructed in the IPA program colored according to genes correlating with circulating leptin levels. Red indicates a positive correlation coefficient and green indicates a negative correlation coefficient. Intensity of the color is related to the strength of the correlation.

Hunger signaling extends beyond these key genes and therefore we constructed a hunger signaling pathway based on expert knowledge and curated databases using Ingenuity Pathway Analysis (IPA) software. Downstream from the leptin receptor (*Lepr*) is the intracellular JAK/STAT signaling pathway, which connects circulating leptin levels to gene expression of *Npy*, *Agrp* and *Pomc*. Insulin and IGF-1 are involved in a parallel nutrient-sensing pathway —the insulin/IGF-1 signaling pathway— and this induces further downstream gene regulation via PI3K and Akt. TNF-α potentially signals via the JAK/STAT signaling pathway via its downstream genes TNF receptor associated factor 4 (*Traf4*) and PTK2 (protein tyrosine kinase 2 beta, *Ptk2b*) which is connected with signal transducer and activator of transcription 3 (*Stat3*) and Janus kinase 2 (*Jak2*) (Fig 4). Downstream from the main hormone receptors there are connections to melanocortin receptors (e.g. *Mc4r* – *Agrp*; *Mcr4* – *Pomc*), dopamine receptors (e.g. *Drd1* – *Pomc*), serotonin receptors (e.g. *5Htr2a* – *Jak2*), thyroid metabolism (e.g. *Thr* – *Htt* – *Cartpt* and *Npy*), metabolism regulation (e.g. *Npy* – *Foxo1* – *Pparg* and *Sirt1*) uncoupling proteins (e.g. *Insr* – *Upc2*) and circadian rhythms (e.g. A*rntl* – *Cartpt*, *Npy* and *Agrp*).

This network highlighted that downstream of the hormone receptors, other hunger related genes werecorrelated with peripheral circulating hormone levels. Leptin correlated with genes involved in JAK/STAT signaling, PI3K/AKT signaling, dopamine receptors, 5-HT receptors, thyroid metabolism and *Arntl* which is involved in circadian rhythms (Figure 4). The correlations between the elements of this hunger signaling pathway and levels of other circulating hormones such as insulin, TNF-α, IGF-1, resistin and IL6 can be found in Figs S1-S5. Principally these athways indicated that lowered IGF-1 was a strong correlate of hunger signaling. In addition, lower levels of TNF-α and insulin showed similar correlation patterns to the decreased levels of leptin. In contrast the pathway.

We also identified several additional hunger related genes that were correlated with circulating hormone levels that were not identified by the IPA database. These included butyrylcholinesterase (*Bche*), prolylcarboxypeptidase (angiotensinase C) (*Prcp*), neurotensin (*Nts*), glucagon-like peptide 1 receptor (*Glp1r*), glutamate receptor ionotropic NMDA3B (*Grin3b*), serum/glucocorticoid regulated kinase 1 (*Sgk1*), bone morphogenetic protein 7 (*Bmp7*) and adiponectin receptor 2 (*Adipor2*) (Table 3).

**Table 3:** Correlations between expression levels of additional hunger related genes and circulating hormones

	Leptin		IGF-1		insulin		TNF-α	
	*r*	p-value	*r*	p-value	*r*	p-value	*r*	p-value
*Bche*	0.707	<0.001	0.632	<0.001				
*Prcp*			0.667	<0.001				
*Nts*			0.622	<0.001	0.465	0.011		
*Glp1r*					0.574	0.001		
*Grin3b*							0.512	0.005
*Sgk1*	−0.815	<0.001	−0.529	0.003			−0.588	0.001
*Bmp7*							−0.496	0.006
*Adipor2*	−0.740	<0.001						

### Graded CR responses involve an upregulation of core clock genes and these are negatively correlated with circulating hormone levels

**Figure 5:**
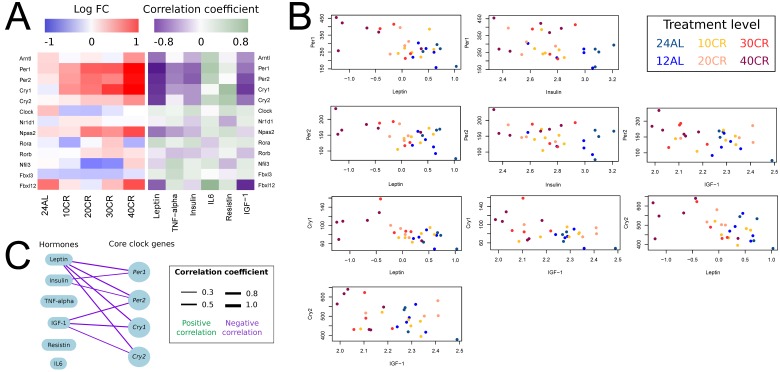
Effect of graded calorie restriction on circulating hormone levels and genes involved in circadian rhythm. (A) Genes involved in circadian rhythm pathways based on their log fold change relative to *ad libitum* feeding for 12h per day (12AL) and their correlation coefficient with circulating hormone levels. Blue indicates down-regulation and red upregulation relative to 12AL. Purple indicates a negative correlation coefficient and green a positive correlation with circulating hormone levels. 10CR, 20CR, 30CR and 40CR refers to 10 %, 20 %, 30 % and 40 % restriction and 24AL refers to 24h *ad libitum* feeding. (B) Expression levels of core clock genes plotted against circulating hormone levels. Those genes with a significant correlation based on p-value < 0.05 are included in the plot. (C) Overview of core clock genes correlating with circulating hormone levels. The lines connecting hormones and genes represent correlations and the width of these lines indicate the strength of the correlation coefficient. Green indicates a positive correlation and purple a negative correlation.

The core clock genes, period circadian clock 1 (*Per1*, log FC 0.630, p-value < 0.001), period circadian clock 2 (*Per2* log FC 0.602, p-value < 0.001) and cryptochrome 1 (photolyase-like) (*Cry1*, log FC 0.640, p-value = 0.001) were significantly upregulated at 40CR relative to 12AL while no significant differences were observed at other CR levels (Fig 5A). Leptin correlated negatively with Per1, Per2, Cry1 and *Cry2*. Insulin correlated negatively with *Per1* and *Per2*; and IGF-1 correlated negatively with *Cry1*, *Cry2*, and *Per2*. No significant correlation was observed between core clock genes and TNF-α, IL6 and resistin (Fig 5B and 5C) (Table 4).

**Table 4:** Correlations between expression levels of key circadian genes and circulating hormones

	leptin		TNF-α		Insulin		IL6		Resistin		IGF-1	
	*r*	p-value	*r*	p-value	*r*	p-value	*r*	p-value	*r*	p-value	*r*	p-value
*Per1*	−0.609	<0.001	−0.352	0.061	−0.402	0.031	0.282	0.138	0.088	0.649	−0.364	0.052
*Per2*	−0.555	0.002	−0.336	0.075	−0.429	0.020	0.242	0.205	0.017	0.929	−0.465	0.011
*Cry1*	−0.540	0.003	−0.266	0.163	−0.275	0.148	0.028	0.887	0.328	0.082	−0.516	0.004
*Cry2*	−0.514	0.004	−0.050	0.796	−0.278	0.145	0.208	0.279	0.279	0.142	−0.371	0.047

**Figure 6:**
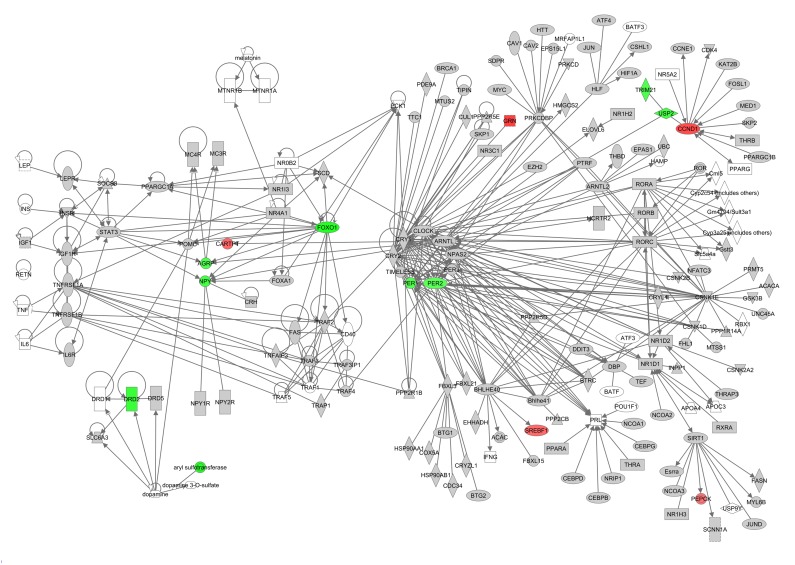
Genes involved in circadian rhythm pathway constructed in the IPA program colored according their correlation with circulating levels of tumor necrosis factor alpha (TNF-α). Red indicates a positive correlation coefficient while green indicates a negative correlation coefficient. Intensity of the color is related to the strength of the correlation.

We also explored how these circadian genes were related to the hunger signaling pathways using IPA. Several direct connections were found between the circadian genes and hunger, including *Arntl, Npy*, *Agrp* and *Cartpt*. However, this network highlighted that the only currently known direct link from hunger to circadian rhythms was via the TNF-α signaling pathway (Fig 6). Circulating levels of TNF-α correlated with *Per2,* but none of the connected genes in the constructed circadian pathway showed a similar correlation. Similar results were found for *Per1*. However, genes downstream from *Arntl* did correlate with TNF-α, such as tripartite motifcontaining 21 (*Trim21*, *r* = −0.360, p-value = 0.055), ubiquitin specific peptidase 2 (*Usp2*, *r*= −0.381, p-value = 0.041) and cyclin D1 (*Ccnd1*, *r* = 0.398, p-value = 0.03). The correlations of the circadian rhythm pathway elements to other circulating hormones such as leptin, insulin, IGF-1, resistin and IL6 can be found in Figs S6-S10.

These pathways indicated that lowered leptin resulted in a higher significance and number of correlating genes compared to other circulating hormones. In addition, lower levels of IGF-1 showed similar correlation patterns to the decreased levels of leptin. The pathway was less correlated with circulating levels of insulin, IL6 and resistin.

### Graded CR phenotypic responses are associated with expression levels of core circadian rhythm genes and hunger genes

**Figure 7:**
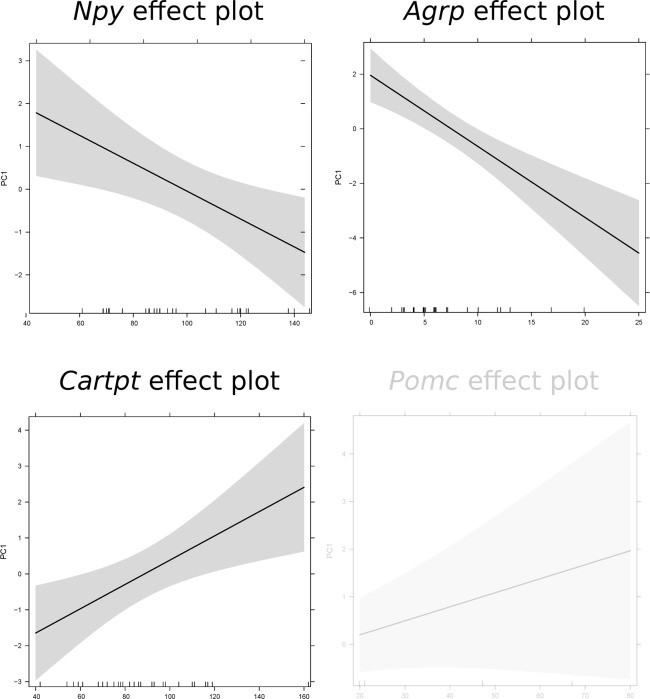
Prediction effect plots of the linear model with eigenvector values of principle component 1 (representing phenotypic responses) and gene expression levels of key hunger genes. A cut-off value of p-value < 0.05 was used to determine significant relationships in these linear models. Non-significance is indicated by light grey.

We previously established that these mice exhibit two phenotypic responses (modulated physical activity patterns [23] and reduced body temperature [22]) to CR throughout the CR study, which can be described as three states [23]: state I : active state with high activity and slightly higher body temperature relative to 12AL; state II: inactive state with low activity and slightly lower body temperature relative to 12AL; and state III: deeply inactive state with even less activity and a large drop in body temperature with occurrence of torpor [23]. We observed a phenotypic shift which involved a decline in the characteristics of these states (decline in body temperature and an economy of movements [23]). We therefore investigated whether the phenotypes described by these states could be associated with the expression level of hunger genes and core clock genes. We initially explored the phenotypic response profile of different groups by applying principal component analysis (PCA). The first two principal components explained ∼80% of the variance and individuals clustered according to CR level. This indicated that CR explained a majority of the variability observed in the phenotypic responses (Fig S11). Movement of state I (the most active state) seemed to drive the separation of 40CR, 30CR and 20CR individuals and AL groups. We further elaborated on the observed separation by analysing if phenotypic responses were associated with a single gene expression or a combination of genes. None of the variance observed in PC2 was significantly explained by gene expression level but variance in PC1 was. A significant proportion of the variance in behavioral responses could be explained by expression levels of 3 of the 4 key hunger genes, with the exception being *Pomc* (linear model (lm) PC1: F(1,31) = 2.272, pvalue = 0.141): *Npy* (F(1,31) = 7.501, p-value = 0.010), *Agrp* (F(1,31) = 25.26, p-value < 0.001) and *Cartpt* (F(1,31) = 6.101, p-value = 0.019) (Fig 7). Phenotypic responses were also significantly explained by the combined expression of these key genes (F(4,28) = 8.385, p-value < 0.001).

**Figure 8:**
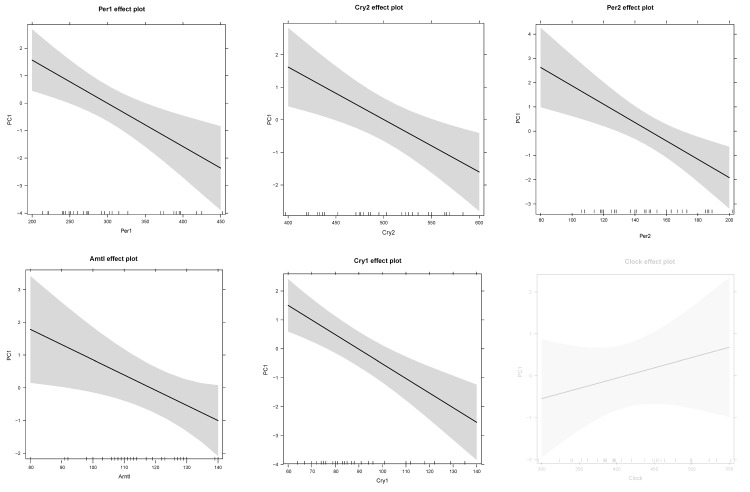
Prediction effect plots of the linear model with eigenvector values of principle component 1 (representing phenotypic responses) and gene expression levels of core clock genes. A cut-off value of p-value < 0.05 was used to determine significant relationships in these linear models. Non-significance is indicated by light grey.

Similar results were found for the 5 core clock genes (lm PC1: F(6,26) = 4.436, p-value < 0.001) and phenotypic responses were significant associated with *Per1* (F(1,31) = 12.67, p-value = 0.001), *Per2* (F(1,31) = 11.47, p-value = 0.002), *Cry1* (F(1,31) = 19.36, p-value < 0.001), *Cry2* (F(1,31) = 9.751, p-value = 0.004) and *Arntl* (F(1,31) = 11, p-value = 0.002) but not on *Clock* (F(1,31) = 0.139, p-value = 0.712) expression levels (Fig 8).

To further elaborate on the significant association between phenotypic responses and gene expression data, we explored the correlations between genes and two distinctive CR response: FAA and mean body temperature (Tb) over the last 20 days of treatment. We found that *Npy* and *Agrp* correlated negatively with Tb and *Cartpt* and *Pomc* (log converted) correlated positively with Tb. *Per1*, *Per2*, *Cry1* and *Cry2* significantly correlated in a negative manner with Tb (Fig S12). We explored if key genes correlated with either FAA or non FAA from the last 20 days. *Npy* and *Agrp* correlated positively with FAA while *Cartpt* and *Pomc* (log converted) correlated negatively (Fig S13). No significant correlation was found between these genes and non FAA (Table 5). Similar results were found for *Per1*, *Per2*, *Cry1* and *Cry2* which correlated positively with FAA but not with non FAA (Fig S14). Furthermore hunger signaling genes in the IPA pathway and additional hunger related genes correlated with FAA (Table S1).

**Table 5:** Correlations between expression levels of key genes and the averaged body temperature (Tb), food anticipatory activity (FAA) and non FAA of the last 20 days

	Tb		FAA		Non FAA	
	r	p-value	r	p-value	r	p-value
*Npy*	−0.446	0.008	0.528	0.002	−0.010	0.956
*Agrp*	−0.669	<0.001	0.585	<0.001	−0.158	0.380
*Pomc*	0.299	0.086	−0.249	0.163	−0.088	0.625
*Cartpt*	0.434	0.010	−0.443	0.010	−0.032	0.861
*Per1*	−0.445	0.008	0.455	0.008	−0.084	0.640
*Per2*	−0.543	0.001	0.569	0.001	0.012	0.948
*Cry1*	−0.558	0.001	0.595	<0.001	−0.269	0.131
*Cry2*	−0.403	0.018	0.361	0.039	−0.256	0.150
*Log_10_Pomc*	0.540	0.001	−0.510	0.003	0.010	0.937

## DISCUSSION

Although previous studies have highlighted a relationship between the extent of restriction and the extent to which lifespan is increased (reviewed in [6,37]), few have investigated the variability in biological responses to a graded increase in CR level [38]. Our previous work has shown that graded calorie restriction influenced body composition, circulating hormone levels, glucose homeostasis, body temperature and phenotypic behavior responses [23,35,36]. Here we anticipated that graded CR would result in a graded transcriptomic response in the hypothalamus. We found that graded CR had an impact on nearly 10% of the hypothalamic genes, including large changes in the hunger signaling and circadian rhythm pathways.

### Graded CR response of hunger related genes and their impact on phenotypic responses

Nutrient-sensing neurons are located in the hypothalamus [39,40]. Neurons in this area, which includes the ventromedial nucleus and the arcuate nucleus, regulate glucose homeostasis, energy balance and body temperature as a response to nutritional deprivation [41,42]. Many studies in *Caenorhabditis elegans* and *Drosophila melanogaster* have shown that these nutrient-sensing neurons can potentially mediate the CR driven increase in longevity, based on its effects on whole body metabolism [43–45]. Neurons in *C. elegans* can influence insulin-like receptor (daf-2) activity via insulin/IGF-1 like signaling hormones, which than influences lifespan as daf-2 mutants are long-lived [46,47]. Furthermore, ablation of insulin-like peptide-producing median neurosecretory cells in the brain of *D. melanogaster*, leads to flies with an extended median and maximum lifespan plus an increased resistance to oxidative stress [45]. These studies suggest that this nutrient-signaling mediated effect might be translatable to mammals as many components of these pathways are highly conserved across different species [48]. The cross talk between metabolism and the hypothalamus is regulated by circulating hormones associated with adipose tissue and liver mass (e.g. leptin and insulin). These hormones target the hypothalamus to control feeding and signal nutritional status, such as nutrient deprivation [49]. Here we highlighted that key components in the hunger signaling pathway were expressed in a manner reflecting elevated hunger: *Npy* and *Agrp* were upregulated and *Pomc* and *Cartpt* were downregulated in response to increasing levels of CR. Furthermore adiponectin receptors (*Adipor2*) correlated negatively with leptin. *Adipor2* co-localizes with *Pomc* and *Npy* in arcuate neurons and is able to increase AMPK phosphorylation in hypothalamic neurons, indicating a role in energy homeostasis [50]. This elevated hunger signaling correlated with decreased circulating levels of insulin, TNF-α, leptin and IGF-1. Circulating levels of insulin and leptin have previously been suggested to play a role in CR-associated longevity and their ability to signal nutritional status could potentially mediate such an effect [19,51]. Supporting this view, *Npy^−/−^* mice exposed to 30% CR showed an abrogated effect on longevity while no differences were observed in lifespans of *Npy^−/−^* AL fed mice [52]. This study also showed CR exposed *Npy^−/−^* mice did not differ in circulating hormone levels such as insulin, IGF-1 and leptin. Theyalso did not differ in transcript levels of *Pomc* and *Argp* compared to CR exposed wild type mice [52]. This previous study indicates that *Npy* plays a key role in linking CR to lifespan and the upregulation of *Npy* under CR could be a major contributor to the CR-mediated increase in lifespan.

The insulin/IGF-1 signaling pathway is evolutionarily conserved and initial evidence that this pathway plays a key role in ageing comes from studies with *C. elegans* [53,54]. Mutants for the daf-2 gene, which leads to a decreased daf-2 signaling, have a lifespan twice as long as their wild type counterparts [46]. Daf-2 is a key gene in the insulin-like signaling pathway in *C. elegans* and requires the activation of forkhead box protein O (daf- 16) [55]. The increase in lifespan by decreased daf-2 signaling suggested a role of reduced insulin signaling in longevity. FIRKO mice, with fat-specific disruption of the insulin receptor gene, have a low fat mass, loss of normal relationship between leptin and bodyweight, protection against hypothalamic lesion-induce obesity and an increase in mean lifespan [56]. Furthermore female insulin receptor substrate 1 null (IRS1^−/−^) mice but not IRS2^−/−^ mice are long-lived and show resistance against age-sensitive markers [57]. However, female IRS2^−/−^ mice do exhibit increased levels of *Npy* and *Agrp* and reduced levels of *Pomc* [58]. These mice are lifelong insulin resistant and have improved health which suggests that other signaling pathways besides reduced insulin signaling contribute. These studies also highlight that decreased insulin signaling is important for low body fat and reduced fat mass is directly related to lower levels of leptin [36]. Under CR, circulating insulin levels were decreased [36], negating ageassociated impaired glucose clearance and insulin resistance. In human studies, healthy centenarians have a preserved insulin action compared to aged subjects, further supporting a role of insulin in longevity [59]. Early work on CR exposed obese ob/ob mice, which are leptin deficient, showed an increase in lifespan of 50 % compared to non-restricted ob/ob mice while levels of adiposity were high [60]. Interestingly NPY deficient ob/ob mice became less obese due to the reduced food intake and had a attenuation of the obesity syndrome which suggests a role for NPY in leptin deficiency [61]. Furthermore, administration of leptin in ob/ob mice leads to increased expression of *Pomc* and reduced levels of *Agrp* [62]. Additionally *Bmp7* correlated negatively with TNF-α and plays a role in appetite regulation, partly mediated by the mTOR pathway [63]. The mTOR pathway is known to play an important role in ageing and recently this pathway and NF-ĸB signaling in the hypothalamus have been inferred to mediate whole body ageing ([64] and reviewed in [65]). Therefore a decrease in nutrient availability, acting via leptin and insulin signaling, is an essential response to CR where downstream genes could potentially mediate behavior changes and longevity [66].

Mice exposed to CR showed specific behavioral changes such as higher levels of activity within the two hours before feeding, known as food anticipatory activity (FAA), while total daily activity was either decreased [21,67–69] or in some cases increased [70,71]. In our study, total daily activity of CR and AL groups were similar after 3 months [20], although FAA was elevated at greater levels of CR and at 40% CR comprised 30% of the total daily activity [20]. Recent studies have shown that FAA is regulated by high levels of *Agrp* and low levels of *Pomc* [12,72,73] and is rapidly reversed by sensory detection of food, which resets the activation state of *Agrp* and *Pomc* neurons induced by hunger signals [12]. We also found a positive correlation between *Agrp* and FAA and a negative correlation with *Pomc* in our data, supporting the suggested roles of *Agrp* and *Pomc* in FAA. *Agrp* is an antagonist able to block the action of alphamelanocyte-stimulating hormone (α-MSH) the melanocortin receptors [74]. The leptin sensitive melanocortin pathway plays a critical role in regulating food seeking behavior and body weight via receptors *Mc3r* and *Mc4r* [75,76]. However, previous work also suggests that *Agrp* mediated feeding does not require the further down-stream melanocortin pathway, and hence that *Agrp* may directly engage feeding circuits and behavior [72]. If *Agrp* was acting via the melanocortin signaling pathway we would expect the key melanocortin receptors Mc3r and Mc4r would correlate with the circulating hormones we measured. Interestingly, neither of the melanocortin receptors (*Mc3r* and *Mc4r*) correlated with the hormones we measured or FAA, supporting the suggestion of an alternative pathway mediating the effect of *Agrp* on behavior [72]. However, we did find a positive correlation between IGF-1 and *Prcp,* and a negative correlation of IGF-1 and FAA, which is involved in the activation of α-MSH [77]. Furthermore α-MSH, leptin and insulin directly induce the gene *Nts* [78], which was also correlated with circulating hormones and negatively with FAA in our dataset.

In other experiments mice exposed to 25 days of 30% CR with the greatest weight loss compared to those with the lowest weight loss showed different neuropeptide profiles with higher expression of *Npy*, *Agrp* and *Mc3r* and lower levels of dopamine D2 receptor (*Drd2*) [79]. The mice with the highest weight loss also exhibited greater hunger as measured by gorging behavior, which potentially drives their elevated FAA [79]. In our study, dopamine receptors (*Drd2* and *Drd5*) correlated positively with leptin, IGF-1, insulin and TNF-α and *Drd5* correlated positively with FAA. Dopamine is involved in motivational and rewarding aspects of food seeking behavior [80]. Insulin and leptin have direct effects on dopamine neuron functions and behavior [81]. Our gene expression results suggested a regulation of changes in feeding behavior such as FAA. Interestingly, during CR *Npy* null mice did not alter behavior or physiological responses but leptin deletion impaired some FAA such as walking and rearing, but not the full spectrum of typical FAA [82]. However, other studies showed that leptin deficient ob/ob mice have increased food seeking behavior before scheduled feeding, which would suggest that FAA is not regulated by leptin [83,84]. Furthermore, in a double mutant MC3R^−/−^ ob/ob mouse model the FAA was preserved and not attenuated by deletion of MC3R [84] which is consistent with lack of correlation between *Mc3r* expression levels and FAA in our dataset. This would indeed suggest an alternative pathway of *Agrp* on behavior than via the melanocortin pathway [72].

Our mice also exhibited a drop in body temperature [35] and the occurrence of torpor at 30CR and 40CR was part of their phenotypic response [23,35]. Previous work has shown that overexpression of UCP2 in the hypothalamus caused local heating near to the temperature sensing component of the hypothalamus leading to a compensatory reduction in body temperature that was associated with increased longevity, suggesting a causal association of longevity to low body temperature [85]. The decrease in body temperature was probably an adaptive mechanism to reduce energy expenditure when nutrient availability was limited [42]. The hunger genes *Agrp* [10] and *Npy* [86,87] have been previously shown to play a role in thermoregulation [88], and correlated negatively with body temperature in our data. Central administration of *Npy* causes hypothermia and a reduced metabolic rate [88] and 12 month old *Agrp^−/−^* mice exhibited an increased body temperature compared to same aged controls [89]. Ghrelin was found to interact with *Npy*/*Argp* neurons through ionotropic glutamate receptors such as *Glp1r* [90], which was correlated with circulating hormone levels in our data. *Npy^−/−^* mice exhibit shallow, aborted torpor bounds in response to CR and ghrelin administration had no effect in these mice, which suggests the effect of ghrelin on torpor is dependent on NPY neurons [91]. Deactivation of the *Ghrl* gene, which encodes ghrelin, might be dependent on *Bche* [92] and we found a reduced expression of *Bche* with lower levels of leptin and IGF-1. During fasting hypothalamic gene expression of *Sgk1* correlates positively with ghrelin [93] and in our data we found a higher expression of this gene with lower levels of leptin, IGF-1 and TNF-α. Although we did not measure ghrelin, other studies show circulating levels of ghrelin are increased under CR [94,95], and its signaling via *Npy* might be responsible for the CR-observed torpor incidence. Interestingly *ob/ob* mice, which are unable to produce leptin, exhibited bouts of torpor when restricted to one meal per day [96], suggesting a role of low leptin in torpor occurrence [97]. This suggests both circulating hormones ghrelin and leptin probably mediate torpor incidence via *Npy* signaling [98], consistent with the correlation of *Npy* levels to body temperature in our mice.

### Graded CR response of circadian rhythm related genes and their impact on phenotypic responses

The phenotypic responses of our CR mice were also associated with the expression of core clock genes, which are not only involved in the regulation of circadian rhythms, but also play an important role in food processing and energy homeostasis [24]. The circadian clock can affect metabolic processes but it is not well understand how these metabolic processes feedback to affect the circadian clock [34]. Our results showed that the core clock genes *Per1*, *Per2* and *Cry1* were upregulated in the hypothalamus in a graded manner in relation to the graded level of restriction and gene expression of these genes was significantly higher at 40CR compared to 12AL. A previous meta-analysis comparing microarray data from CR restricted mice with AL controls also identified circadian rhythms among the most upregulated biological processes in several different tissues, with *Per2* being the most significant [99]. *Per1* and *Per2* genes might play a role in reducing the mortality rates under CR. They have tumor suppression activity [100], and the decreased tumor incidence is one of the major factors in CR mice leading to increased lifespan [101]. The circadian network we constructed highlighted that the only direct link currently known between hunger and circadian rhythms was via the TNF-α signaling pathway. Circulating TNF-α levels were reduced under CR [36] and found here to correlate with high expression levels of *Per* genes. This is in agreement with previous studies where high levels of TNF-α were found to suppress expression of the *Per* genes [102,103], which would lead to an attenuation of clock genes and disruption of the circadian clock. Furthermore, disruption of the circadian clock is also associated with ageing and includes a reduced amplification of clock gene expression and desynchronisation of physiological rhythms [33]. CR is able to synchronise the circadian clock and these changes might be an important mediator for longevity in CR mice [24]. In agreement with previous studies, our results suggest that under CR, mice are protected against ageingassociated desynchronisation [33,99]. Other studies have shown that mice fed *ad libitum* for 24 hours are prone to develop obesity, and have attenuated diurnal feeding rhythms [104,105]. When food is restricted to 12 hours, these animals gain less weight than those whose food was available over 24 hours, even when calorie intake is held constant [104,105]. Furthermore, mice fed 12 hours *ad libitum* were protected against obesity, hyperinsulinemia, inflammation and metabolic disorders compared to the group fed *ad libitum* for 24 hours [104,105]. In contrast, in our study the two AL groups (one given food continuously and the other restricted to food for 12h per day) did not differ significantly in body weight, body composition, leptin, insulin, TNF-α levels or glucose tolerance [35,36], but did display different transcriptomic profiles. However, no significant differences were observed in clock gene expression between the two AL groups, which suggests no significant impact on core clock genes after three months of 12h restricted feeding.

The link of leptin signaling to the circadian clock mechanisms is a yet undescribed pathway in the IPA software and our data would suggest an association between hunger-signaling via leptin and circadian rhythms. In obese ob/ob mice clock genes in the hypothalamus were not affected, but in liver and adipose tissue these genes are substantially damped which suggests an impairment of peripheral, but not central clock genes. Four weeks of 50% CR was unable to improve peripheral clock function in these mice but administration of leptin did, suggesting a role of leptin in impaired circadian rhythms [106]. In vitro experiments of isolated SCN of rats show a dosedependent response to leptin suggesting leptin can modulate circadian rhythms in the hypothalamus [107] and probably by direct modulation of electrical properties of the SCN neurons [108]. Furthermore ob/ob mice have an altered photic synchronization response and acute leptin treatment normalised this. Leptininduced phosphorylated STAT3 was modulated by light in the arcuate nucleus which suggests an indirect regulation of leptin on the SCN [109]. The JAK/STAT signaling pathway is an import part of leptin signaling and contributes to hunger regulation. The link between leptin, STAT3 and circadian rhythms has not been extensively described but is not a novelty [110,111]. Jet lagged *Per1/Per2* KO mice exhibit low levels of STAT3 expression and high plasma leptin levels while *Cry1/Cry2* KO mice have low leptin levels with high levels of STAT3. Interestingly jet lagged *Per1/Per2* KO mice exhibited leptin resistance due to the loss of STAT3 activation in POMC neurons [112]. Further characterising of this link might elaborate the complex mechanisms induced by CR responses and further downstream beneficial effects.

Hunger signaling, circadian rhythms and their downstream effects are far more complex than the results described here. Although limited by using a knowledge based signaling network, we were able to gain insights into the potential mechanisms underpinning the action of CR. Associations between gene expression and physiological outcomes such as body temperature and food anticipatory activity established by linear models and correlations are obviously only descriptive and causality cannot be assumed. Nevertheless these individual mice have been subjected to an unprecedented level of phenotyping allowing us to tie together the complex transcriptomic changes to alterations in body composition, circulating hormones and physiological outcomes. Future KO studies and manipulations studies would aid in establishing causality between circulating hormones, gene expression and physiological outcomes and further elaborate on the hunger signaling pathways and its downstream effect on longevity. Overall, our study has demonstrated that increasing levels of CR lead to a graded expression of genes involved in both hunger signaling and circadian rhythms. The expression of genes in these pathways were correlated with circulating levels of leptin, insulin, TNF-α and IGF-1, but not resistin or IL-6. We also demonstrated the phenotypic responses to CR (body temperature and physical activity) were significantly associated with the key hunger and core clock genes. Our results suggest that under CR modulation of the hunger and circadian signaling pathways, in response to altered levels of circulating hormones, drive some of the key phenotypic outcomes, such as activity and body temperature, which are probably important components of the longevity effects of CR.

## MATERIALS AND METHODS

To create a systems level description of graded CR responses, we performed a three month graded CR study on male C57BL/6 mice. Behavioral, physiological and molecular information from various tissues of individual mice were collected, including body temperature, physical activity, 24-hour energy expenditure, oxidative damage, behavior data, body composition, resting metabolic rate, circulating hormones and adipokines, metabolomics, proteomics and transcriptomics. In this study, whole transcriptome sequencing or RNA-seq was used to identify differentially expressed genes (DEGs) in the hypothalamus across different CR levels and to assess the role of circulating hormones in the transcriptomic response to graded CR, and the correlations between these transcripts and various behavioral phenotypes.

### Animals and experimental manipulations

All procedures were approved by the University of Aberdeen ethical approval committee and carried out under the Animals (Scientific Procedures) Act 1986 Home Office license (PPL 60/4366 held by SEM). Forty nine male C57BL/6 mice (*Mus musculus*) purchased from Charles River (Ormiston, UK) were individually housed and free access to water was provided. Mice were exposed to 12 hour dark/light cycle (lights on at 0630h) and body mass and food intake were recorded daily, immediately prior to nocturnal feeding. At 20 weeks of age (resembling early adulthood in humans), mice were randomly allocated into 6 different treatment groups: 24h *ad libitum* intake (24AL) (n=8), 12AL intake (n=8), 10 CR (n=8), 20CR (n=8), 30CR (n=8) and 40CR (n=9). Mice in 12AL group were fed *ad libitum* for 12h during the dark period and 40CR indicates 40% lower calories than their own individual intakes measured over a baseline period of 14 days prior to introducing CR.

The 12AL control group was used in the study design as control against obesity and all pairwise analysis is relative to 12AL. Animals fed completely *ad libitum* (i.e., having 24 hours access to food) may overfeed, become overweight and CR associated changes compared to 24AL are therefore most likely to reflect the anti-obesity effect of CR [6,113]. To address this issue, 12AL was set as a reference and graded levels of CR were introduced to investigate a potential graded response. To avoid potentially confounding factors interfering with the circadian rhythm and hormone regulations, all mice were culled between 1400 and 1800, prior to lights out. Detailed information on overall study design, diet composition and detailed rationale are described elsewhere [35].

### RNA isolation, cDNA synthesis and RNA sequencing

After culling by a terminal CO2 overdose, brains were removed, weighed and frozen in isopentane over dry ice and stored at −80°C for RNA isolation. The hypothalamus was carefully dissected at a later stage and RNA was isolated by homogenizing in Tri-Reagent (Sigma Aldrich, UK) according to manufacturer's instructions. Prior to RNA quantification using the Agilent RNA 6000 Nano Kit, samples were denatured at 65°C.

Due to the very small size of the hypothalamus, some samples did not contain sufficient quantity of high quality RNA. In total, the RNA of 37 individual mice (12 h AL n=6, 24 h AL n=6, 10 % CR n=7, 20 % CR n=5, 30 % CR n=5, 40 % CR n=8) was successful isolated and sent to Beijing Genomic Institute (BGI, Hong Kong) for RNA sequencing. Library preparation was conducted by enriching total RNA by using oligo(dT) magnetic beads. Fragmentation buffer was added to obtain short fragments from the RNA. The mRNA was used as a template for the random hexamer primers, which synthesize the first strand of cDNA. The second strand was synthesized by adding buffer dNTPs, RNase and DNA polymerase. A QiaQuick PCR extraction kit was used to purify the double stranded cDNA and washed with EB buffer for end repair and single nucleotide A addition. The fragments were ligated with sequencing adaptors, purified using agarose gel-electrophoresis and enriched by PCR amplification. As a quality control step, an Agilent 2100 Bioanaylzer and ABI StepOnePlus Real-Time PCR System were used to qualify and quantify of the sample library. The library products were sequenced using an Illumina Hiseq 2000, resulting in 50 bp single end reads (standard protocol BGI, Hong Kong). Standard primers and barcodes developed by BGI were used.

### Alignment to the reference genome

Prior to alignment to the reference genome, FASTQ files were quality controlled to identify the presence of adaptors or low quality sequences using fastQC (www.bioinformatics. http://bbsrc.ac.uk/projects/fastqc/). To ensure a high sequence quality, the reads were trimmed with a cut-off phred score of 28 using Trimmomatic [114]. Reads were aligned to the reference genome obtained from the National Center for Biotechnology Information (NCBI) database (*Mus musculus*, version MGSCv37, 2010/09/23, http://www.ncbi.nlm.nih.gov/assembly/165668/). The reference genome was indexed using Bowtie2 [115] and reads aligned with Tophat2 [116] using default settings. Of the 465,857,891 reads 453,726,674 (97.4%) were successfully aligned to the reference genome and 10.8 % contained multi mapped reads. These were removed using the Sequence Alignment/Map (SAM) tools [117] before proceeding to quantifying the reads. The number of reads aligning to a single feature (genes containing exons) was determined using HTSeqcount [118] by identifying how many reads mapped onto a single feature (genes containing exons).

One animal (20 % CR group) was excluded from further analysis based on having > 20 % multi-mapped reads when aligned to the reference transcriptome, suggesting a problem with the sequencing technology. This mouse did not show an abnormal response in other aspects of its phenotype.

### Analytical procedure

To remove any genes that exhibited no or a very low number of mapped reads only genes that had more than 1 count per million in at least 4 samples across all treatments were retained for further analysis. This resulted in a total of 14,013 unique genes. Read counts were normalized using the trimmed mean of M values (TMM normalization) [119] to account for highly expressed genes consuming substantial proportion of the total library size. This composition effect would cause remaining genes to be under sampled [120]. Differential gene expression was modelled using the edgeR package [120] in R (version 3.1.2) [121] and pairwise comparisons were conducted between 12AL and each level of CR. To control for type I error, Benjamini Hochberg adjusted p-value was used (5 %FDR) [122]. Significant genes were identified based on a cut off p-value < 0.05 and an absolute log fold change (log FC) > 0.5.

### Biological interpretation

DEGs based on a likelihood ratio test (LRT, p-value < 0.05) were analyzed with Gene Set Enrichment Analysis (GSEA) software using Gene Ontology biological processes gene set (c5.bp.v5.0.symbols) (www.broadinstitute.org/gsea/downloads.jsp) [123]. The genes were pre-ranked according to their p-value with most significant genes having the highest pre-ranked value. Gene set size filters (min=5, max=800) resulted in filtering out 20 of the 825 gene sets. The remaining gene sets were used in the analysis. A total of 22 pathways resulted in a pvalue < 0.05. The enrichment scores of the biological processes were not taken into consideration as they only represent p-values and not log FC. Output files generated by GSEA were loaded into Cytoscape using the plugin enrichment map (settings: p-value cut off < 0.05, similarity cut off 0.5) and biological processes were visualized as a network [124,125]. Pathway enrichment for 24AL vs 12AL was identified using a similar methodology as above but using the KEGG pathways (c2.cp.kegg.v5.0.symbols) which resulted in filtering out 4 of the 186 gene sets. A total of 36 pathways had a p-value < 0.05.

Data files with log FC per gene for each CR level relative to 12AL were further analyzed using the IPA program by using the option core analysis (Ingenuity® Systems, http://www.ingenuity.com). Pathways and transcription factors were identified based on the significant regulation of their target genes (cut-off pvalue < 0.05 and absolute log FC > 0.5).

Circulating hormone levels (methods and data described in [36]) were correlated with each gene and each individual using Pearson correlations conducted in the statistical environment R (version 3.1.2) [121]. These correlations were used in IPA to map onto the hunger signaling pathway and circadian rhythm pathway. The hunger signaling and circadian rhythm pathways were constructed in the IPA program, which uses literature and expert knowledge-based approaches.

Behavioral phenotypes were determined by hidden Markov models (HMM) for each mouse based on activity and body temperature (methods described in [23]). We showed that mice showed three states: (1) state I: active state. Mice had a higher activity and a slightly higher body temperature; (2) state II: inactive state. Mice moved less and had a slightly lower body temperature; (3) state III: deeply inactive. Mice in a torpor state moved even less and displayed a large drop in body temperature. These three states were analyzed using principle component analysis (PCA). Linear models were constructed to identify significant relationships between eigenvector values from PC1 and PC2 (representing phenotypic responses) and for each key hunger and clock gene separately and then for all genes included. A cut-off value of p-value < 0.05 was used to determine significant relationships in these linear models. Both PCA and linear modeling was conducted in the statistical environment R (version 3.1.2) [121].

The averaged body temperature of the last 20 days of treatment (methods and data described in [36]) correlated with each gene and each individual using Pearson correlations conducted in the statistical environment R (version 3.1.2) [121]. A similar approach was taken for food anticipatory activity. Total physical activity levels of the last 20 days of treatment was separated into food anticipatory activity and nonfood anticipatory activity. The values were averaged and correlated with each gene and each individual using Pearson correlations conducted in the statistical environment R (version 3.1.2) [121].

## SUPPLEMENTARY RESULTS












