# Supplementary material for: The effects of graded levels of calorie restriction: VI. Impact of short-term graded calorie restriction on transcriptomic responses of the hypothalamic hunger and circadian signaling pathways
Source: Aging (Albany NY). 2016 Feb 23;8(4):642–61. doi: 10.18632/aging.100895 (PMC4925820; doi:10.18632/aging.100895)
Supplement: Supplementary file 1 [file aging-08-642-s001.pdf]

SUPPLEMENTARY RESULTS

Comparison between 24h ad libitum (24AL) feeding and 12h ad libitum (12AL) feeding

In total 103 genes were significantly differentially expressed at 24AL relative to 12AL (p-value < 0.05, absolute log fold change (log FC) > 0.5); 34 down regulated in 24AL and 69 up regulated in 24AL. Pathway analysis (Gene Set Enrichment Analysis) of the transcriptomic response (n= 14013 genes) to 24AL identified 36 pathways to be significant altered relative to 12AL; of which 13 were up regulated and 23 were down regulated (p-value < 0.05). Metabolism related pathways (i.e. amino acid metabolism, carbohydrate metabolism, energy metabolism and lipid metabolism) were down regulated for the 24AL group relative to 12AL. One of these pathways was 'sphingolipid metabolism'. The two pathways related to immune system 'RIG I like receptor signalling pathway' and 'instestinal immune network for IGA production' were up regulated relative to 12AL; while 'apoptosis' (involved in cell growth an death) was down regulated. Signalling pathways such as 'calcium signalling pathway', 'ECM receptor interaction', 'neuroactive ligand receptor interaction' and 'cell adhesion molecules (CAMs) were up regulated. The pathway 'DNA replication', which is involved in replication and repair, was down regulated. Other pathways involved in genetic information processing were also down regulated (Figure S15).

CR responses of gene expression, transcription factors and pathways relative to 12AL

The number of significantly differently expressed genes at each level of CR relative to 12AL increased in a graded manner: 117, 152, 133 and 385 respectively. In total 80 genes were exclusively expressed at 10CR, 110 at 20CR, 69 at 30CR at 294 at 40CR. Only five genes that were differentially expressed relative to 12AL were common to all four levels of restriction (Figure S16): Npy, nidogen 2 (Nid2), deleted in lymphocytic leukemia 7 (Dleu7), transmembrane protein 232 (Tmem232) and fibrinogen-like protein 2 (Fgl2). Pathway analysis of the differentially expressed genes at each CR level revealed a total of 16 pathways exclusively regulated at 10CR, 49 at 20CR, 16 at 30CR and 70 at 40CR. A specific CR response was seen on 21 pathways but different genes were involved at different levels of CR (Figure S16B). Based on the regulation of downstream genes a total of 26 transcription factors were identified to be exclusively regulated at 10CR, 53 at 20CR, 17 at 30CR and 183 at 40CR. Over the four levels of CR we observed NF-κB (complex) to be inhibited across all CR levels (Figure S16C).

SUPPLEMENTARY DATA

Please browse Full text version to see Supplementary Figures and Table of this manuscript.

Supplementary Figure Legends

Fig S1. Genes involved in hunger pathway constructed in the IPA program colored according their correlation with circulating levels of insulin-like growth factor 1 (IGF-1). Red indicates a positive correlation coefficient while green indicates a negative correlation coefficient. Intensity of the color is related to the strength of the correlation.

Fig S2. Genes involved in hunger pathway constructed in the IPA program colored according their correlation with circulating levels of interleukin 6 (IL6). Red indicates a positive correlation coefficient while green indicates a negative correlation coefficient. Intensity of the color is related to the strength of the correlation.

Fig S3. Genes involved in hunger pathway constructed in the IPA program colored according their correlation with circulating levels of insulin. Red indicates a positive correlation coefficient while green indicates a negative correlation coefficient. Intensity of the color is related to the strength of the correlation.

Fig S4. Genes involved in hunger pathway constructed in the IPA program colored according their correlation with circulating levels of resistin. Red indicates a positive correlation coefficient while green indicates a negative correlation coefficient. Intensity of the color is related to the strength of the correlation.

Fig S5. Genes involved in hunger pathway constructed in the IPA program colored according their correlation with circulating levels of tumor necrosis factor alpha (TNF-α). Red indicates a positive correlation coefficient while green indicates a negative correlation coefficient. Intensity of the color is related to the strength of the correlation.

Fig S6. Genes involved in circadian rhythm pathway constructed in the IPA program colored according their correlation with circulating levels of insulin-like growth factor 1 (IGF-1). Red indicates a positive correlation coefficient while green indicates a negative correlation coefficient. Intensity of the color is related to the strength of the correlation.

Fig S7. Genes involved in circadian rhythm pathway constructed in the IPA program colored according their correlation with circulating levels of interleukin 6 (IL6). Red indicates a positive correlation coefficient while green indicates a negative correlation coefficient. Intensity of the color is related to the strength of the correlation.

Fig S8. Genes involved in circadian rhythm pathway constructed in the IPA program colored according their correlation with circulating levels of insulin. Red indicates a positive correlation coefficient while green indicates a negative correlation coefficient. Intensity of the color is related to the strength of the correlation.

Fig S9. Genes involved in circadian rhythm pathway constructed in the IPA program colored according their correlation with circulating levels of resistin. Red indicates a positive correlation coefficient while green indicates a negative correlation coefficient. Intensity of the color is related to the strength of the correlation.

Fig S10. Genes involved in circadian rhythm pathway constructed in the IPA program colored according their correlation with circulating levels of leptin. Red indicates a positive correlation coefficient while green indicates a negative correlation coefficient. Intensity of the color is related to the strength of the correlation.

Fig S11. Principal component analysis demonstrating the effect of graded CR on behavioral phenotype. Mice exhibited three states: active high, inactive and deeply inactive. These states are indicated on the graph by high, mid and low respectively. Behavioral phenotype was measured by movement and temperature indicated by mv and temp respectively. 10CR, 20CR, 30CR and 40CR refer respectively to 10 %, 20 %, 30 % and 40 % restriction, 12AL refers to 12h *ad libitum* feeding and 24AL refers to 24h *ad libitum* feeding.

Fig S12. Correlations between expression levels of key hunger and circadian genes and the average body temperature at the last 20 days of treatment. Expression values of *Pomc* were log transformed to the base 10. 10CR, 20CR, 30CR and 40CR refer respectively to 10 %, 20 %, 30 % and 40 % restriction, 12AL refers to 12h *ad libitum* feeding and 24AL refers to 24h *ad libitum* feeding.

Fig S13. Correlations between expression levels of key hunger genes and the average food anticipatory active (FAA) and non-FAA at the last 20 days of treatment. Expression values of *Pomc* were log transformed to the base 10. 10CR, 20CR, 30CR and 40CR refer respectively to 10 %, 20 %, 30 % and 40 % restriction, 12AL refers to 12h *ad libitum* feeding and 24AL refers to 24h *ad libitum* feeding.

Fig S14. Correlations between expression levels of key circadian genes and the average food anticipatory active (FAA) and non-FAA at the last 20 days of treatment. 10CR, 20CR, 30CR and 40CR refer respectively to 10 %, 20 %, 30 % and 40 % restriction, 12AL refers to 12h *ad libitum* feeding and 24AL refers to 24h *ad libitum* feeding.

Figure S15. Differentially regulated pathways after three months of 24 hours *ad libitum* (24AL) feeding relative to 12 *hours ad libitum* feeding (12AL) visualized by Enrichment map (Cytoscape). The nodes represent pathways, and edges represent overlap between genes in the pathways. The color of the nodes represents the significance according to the NES enrichment score (blue: down regulated, red: up regulated). The size of the nodes corresponds to the size of the gene set. The width of edges is based on similarity coefficients (> 0.5) between the nodes, derived from the overlap of the gene set underlying the pathways.

Fig S16. Calorie restriction (CR) responses of gene expression, transcription factors and pathways relative to 12 hours *ad libitum* feeding (12AL). (A) differentially expressed genes (DEG) for each level of CR relative to 12AL. (B) canonical pathways for each level of calorie restriction (CR) (data generated from IPA). (C) transcription factors for each level of CR (data generated from IPA) 10CR, 20CR,30CR and 40CR refer respectively to 10 %, 20 %, 30 % and 40 % restriction, 12ALrefers to 12h *ad libitum* feeding and 24AL refers to 24h *ad libitum* feeding.

Supplementary Table Legends

Table S1. Correlations between expression levels of hunger signaling genes and food anticipatory activity (FAA) and non FAA of the last 20 days.
