# Supplementary figures and images for: The effects of graded levels of calorie restriction: VI. Impact of short-term graded calorie restriction on transcriptomic responses of the hypothalamic hunger and circadian signaling pathways
Source: Aging (Albany NY). 2016 Feb 23;8(4):642–61. doi: 10.18632/aging.100895 (PMC4925820; doi:10.18632/aging.100895)

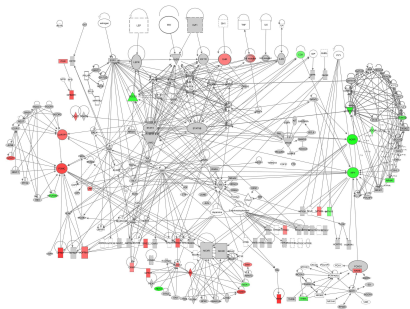

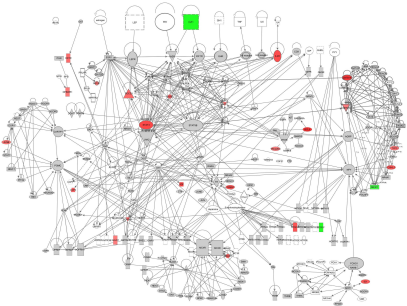

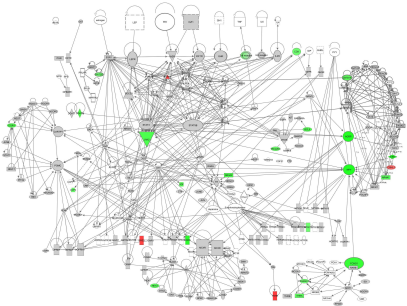

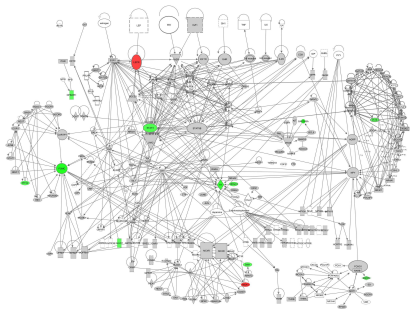

Supplement: Supplementary file 2 [file aging-08-642-s002.pdf]

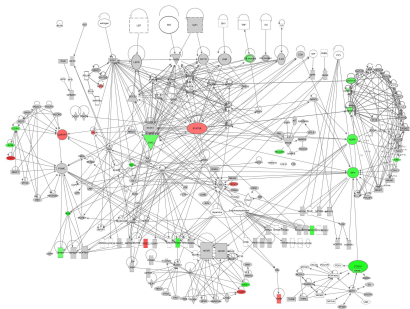

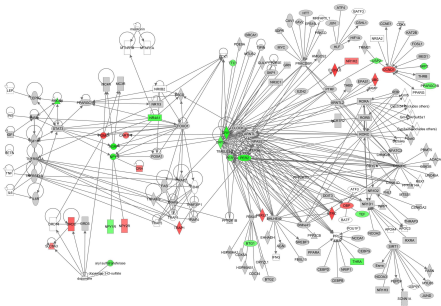

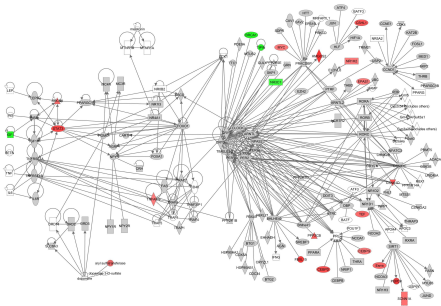

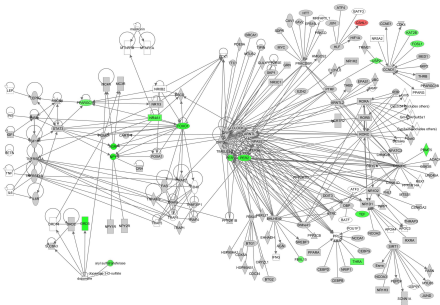

Supplement: Supplementary file 3 [file aging-08-642-s003.pdf]

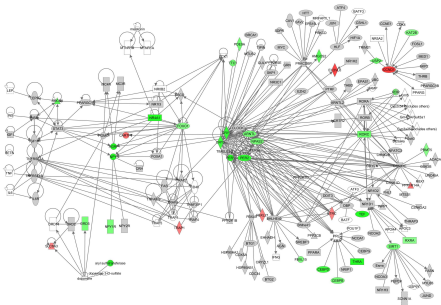

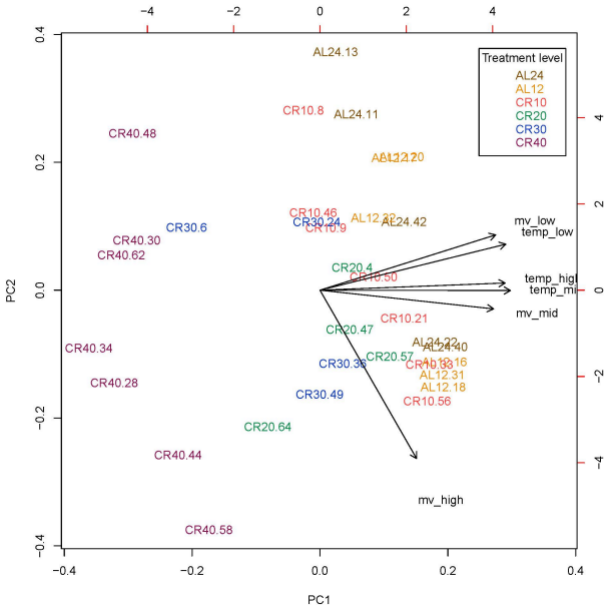

Treatment  
level

24AL  
12AL  
10CR  
20CR  
30CR  
40CR

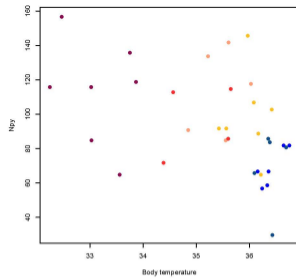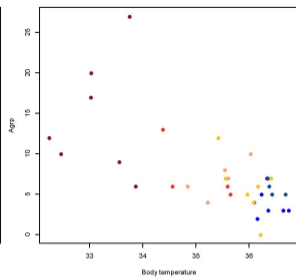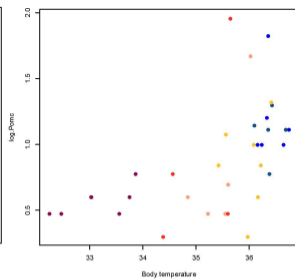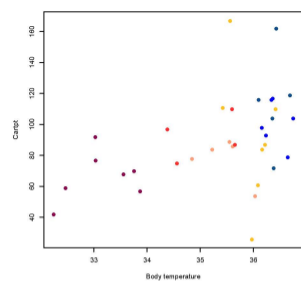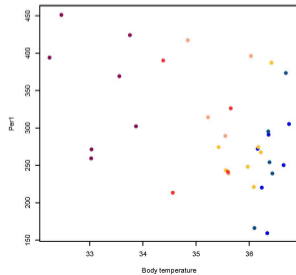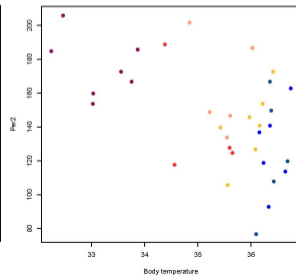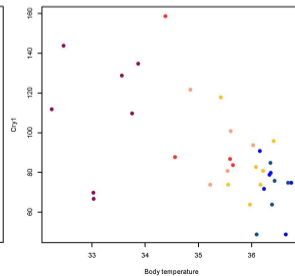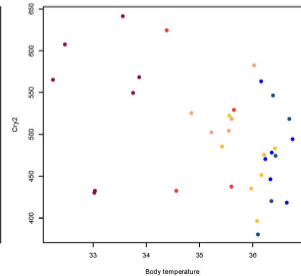

Supplement: Supplementary file 4 [file aging-08-642-s004.pdf]

Treatment  
level

24AL

12AL

10CR

20CR

30CR

40CR

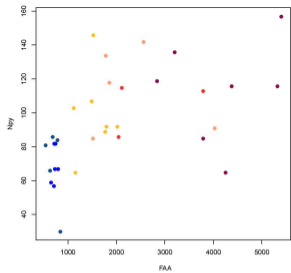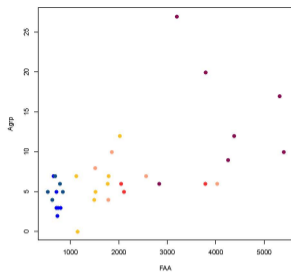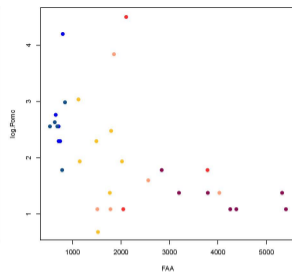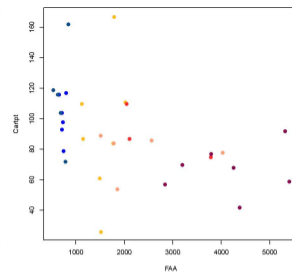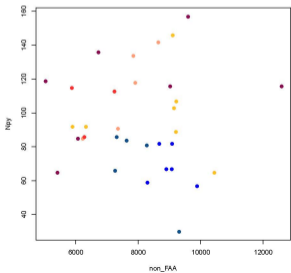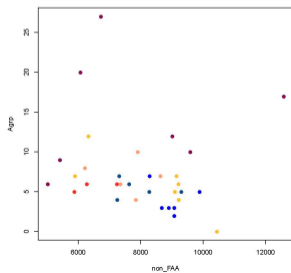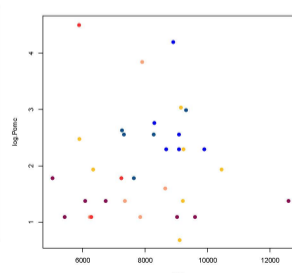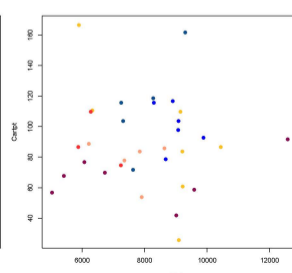

Treatment  
level

24AL

12AL

10CR

20CR

30CR

40CR

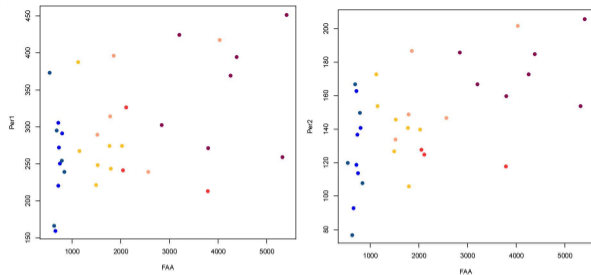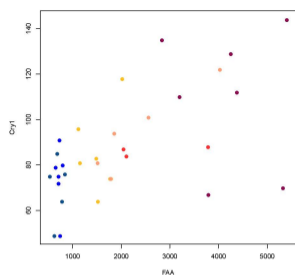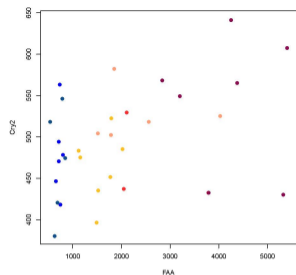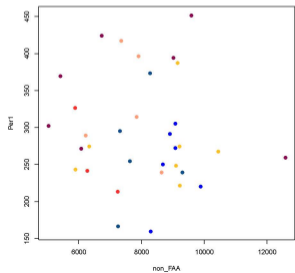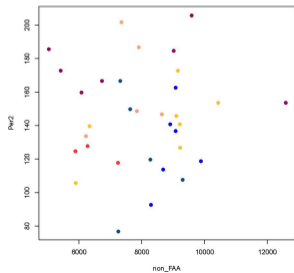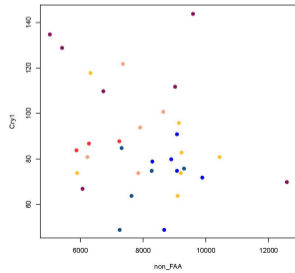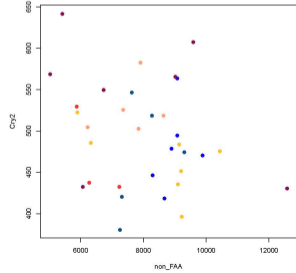

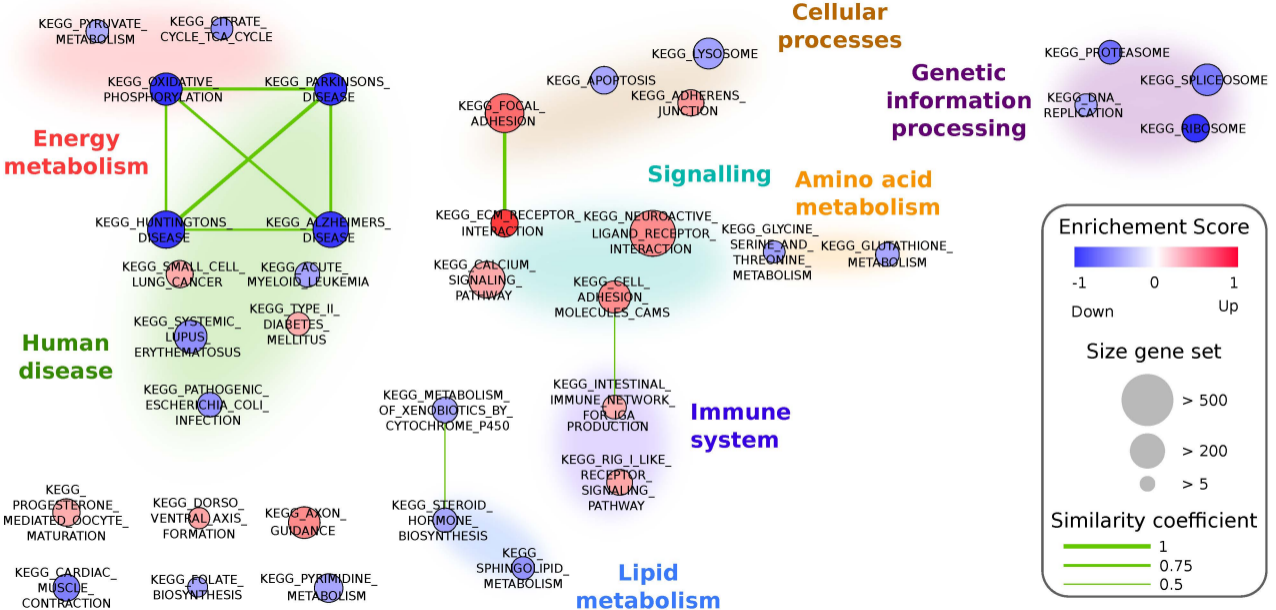

A

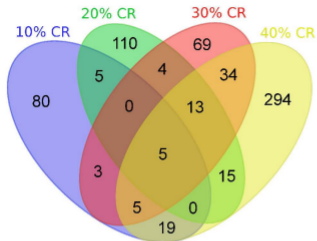

B

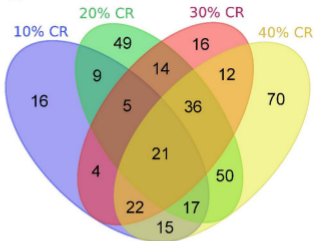

C

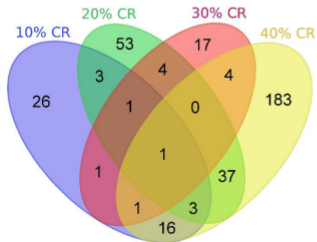

Supplement: Supplementary file 5 [file aging-08-642-s005.pdf]
