# Supplementary material for: The effects of graded levels of calorie restriction: VI. Impact of short-term graded calorie restriction on transcriptomic responses of the hypothalamic hunger and circadian signaling pathways
Source: Aging (Albany NY). 2016 Feb 23;8(4):642–61. doi: 10.18632/aging.100895 (PMC4925820; doi:10.18632/aging.100895)
Supplement: Supplementary file 6 [file aging-08-642-s006.docx]

Table S1. Correlations between expression levels of hunger signalling genes and food anticipatory activity (FAA) and non FAA of the last 20 days.

|  |  | FAA | | Non-FAA | |
| --- | --- | --- | --- | --- | --- |
|  |  | r | p-value | r | p-value |
| Additional hunger genes | *Bche* | -0.559 | 0.001 | 0.169 | 0.346 |
|  | *Prcp* | -0.506 | 0.003 | 0.114 | 0.527 |
|  | *Nts* | -0.411 | 0.018 | 0.045 | 0.805 |
|  | *Glp1r* | -0.292 | 0.100 | 0.019 | 0.917 |
|  | *Grin3b* | -0.069 | 0.702 | -0.024 | 0.894 |
|  | *Sgk1* | 0.772 | <0.001 | 0.063 | 0.726 |
|  | *Bmp7* | -0.046 | 0.801 | 0.004 | 0.982 |
| Hunger signalling pathway IPA | *Adipor2* | 0.683 | <0.001 | 0.087 | 0.629 |
|  | *Adrbk1* | 0.052 | 0.776 | 0.041 | 0.822 |
|  | *Agt* | 0.110 | 0.543 | -0.195 | 0.276 |
|  | *Akt1* | -0.038 | 0.832 | -0.037 | 0.837 |
|  | *Arnt2* | -0.094 | 0.602 | -0.167 | 0.353 |
|  | *Arntl* | 0.229 | 0.199 | -0.073 | 0.688 |
|  | *Atrnl1* | -0.431 | 0.012 | -0.119 | 0.509 |
|  | *Avp* | -0.286 | 0.106 | -0.018 | 0.921 |
|  | *Bcl6* | 0.154 | 0.391 | -0.189 | 0.292 |
|  | *Brs3* | -0.320 | 0.070 | -0.304 | 0.085 |
|  | *Cck* | 0.209 | 0.243 | 0.076 | 0.673 |
|  | *Ccnd1* | -0.714 | <0.001 | 0.114 | 0.529 |
|  | *Cct6a* | -0.108 | 0.550 | -0.111 | 0.540 |
|  | *Cdkn1a* | 0.600 | <0.001 | 0.123 | 0.496 |
|  | *Cdkn1b* | -0.187 | 0.297 | 0.190 | 0.290 |
|  | *Cnr1* | -0.167 | 0.353 | 0.054 | 0.765 |
|  | *Crem* | -0.018 | 0.920 | 0.182 | 0.311 |
|  | *Crh* | -0.295 | 0.096 | 0.016 | 0.928 |
|  | *Dgat1* | -0.502 | 0.003 | 0.134 | 0.457 |
|  | *Dgat2* | 0.373 | 0.032 | -0.162 | 0.368 |
|  | *Drd2* | -0.080 | 0.659 | -0.049 | 0.787 |
|  | *Drd5* | 0.393 | 0.024 | -0.193 | 0.283 |
|  | *Egfr* | -0.270 | 0.129 | 0.307 | 0.082 |
|  | *Egr1* | 0.184 | 0.306 | 0.077 | 0.669 |
|  | *En1* | -0.284 | 0.109 | -0.081 | 0.655 |
|  | *Ep300* | -0.187 | 0.297 | 0.015 | 0.932 |
|  | *Esr1* | -0.360 | 0.040 | -0.100 | 0.581 |
|  | *Fap* | 0.302 | 0.088 | 0.198 | 0.269 |
|  | *Foxo1* | 0.297 | 0.094 | -0.038 | 0.832 |
|  | *Fto* | -0.333 | 0.059 | -0.258 | 0.147 |
|  | *Gadd45a* | 0.102 | 0.572 | 0.156 | 0.387 |
|  | *Gal* | -0.245 | 0.169 | -0.027 | 0.880 |
|  | *Galr1* | -0.147 | 0.416 | -0.109 | 0.544 |
|  | *Gata2* | 0.190 | 0.290 | -0.231 | 0.196 |
|  | *Ghr* | -0.394 | 0.023 | 0.077 | 0.668 |
|  | *Ghsr* | -0.059 | 0.743 | 0.052 | 0.775 |
|  | *Gipr* | 0.198 | 0.270 | -0.085 | 0.639 |
|  | *Gnas* | -0.329 | 0.062 | -0.254 | 0.153 |
|  | *Gpr37* | -0.391 | 0.024 | -0.144 | 0.425 |
|  | *Grb10* | -0.309 | 0.080 | -0.209 | 0.243 |
|  | *Grip1* | -0.216 | 0.227 | -0.204 | 0.254 |
|  | *Grm1* | -0.373 | 0.033 | -0.255 | 0.152 |
|  | *Hcrt* | -0.137 | 0.448 | -0.141 | 0.434 |
|  | *Hcrtr1* | -0.250 | 0.160 | -0.148 | 0.411 |
|  | *Hcrtr2* | 0.013 | 0.941 | 0.189 | 0.293 |
|  | *Hdac1* | -0.373 | 0.032 | -0.270 | 0.128 |
|  | *Hdac2* | -0.098 | 0.589 | -0.126 | 0.484 |
|  | *Hdac3* | -0.334 | 0.057 | 0.033 | 0.856 |
|  | *Hdac4* | -0.325 | 0.065 | 0.125 | 0.488 |
|  | *Hes1* | -0.263 | 0.139 | 0.161 | 0.371 |
|  | *Hey2* | 0.302 | 0.088 | 0.076 | 0.675 |
|  | *Htr1a* | -0.360 | 0.040 | 0.017 | 0.925 |
|  | *Htr1b* | -0.056 | 0.755 | 0.126 | 0.483 |
|  | *Htr1d* | -0.159 | 0.378 | -0.094 | 0.601 |
|  | *Htr1f* | -0.006 | 0.974 | 0.218 | 0.224 |
|  | *Htr2a* | -0.051 | 0.779 | 0.120 | 0.506 |
|  | *Htr2c* | -0.226 | 0.207 | -0.252 | 0.157 |
|  | *Htr3a* | -0.174 | 0.333 | 0.046 | 0.801 |
|  | *Htr4* | -0.347 | 0.048 | 0.250 | 0.160 |
|  | *Htr5a* | -0.176 | 0.328 | -0.211 | 0.239 |
|  | *Htr5b* | 0.268 | 0.131 | 0.170 | 0.343 |
|  | *Htt* | -0.312 | 0.077 | 0.026 | 0.885 |
|  | *Id1* | 0.042 | 0.815 | 0.035 | 0.845 |
|  | *Igf1* | -0.156 | 0.387 | 0.294 | 0.097 |
|  | *Igf1r* | -0.261 | 0.143 | 0.037 | 0.838 |
|  | *Il1rap* | 0.146 | 0.416 | -0.117 | 0.518 |
|  | *Insr* | -0.047 | 0.796 | -0.038 | 0.834 |
|  | *Irs1* | -0.003 | 0.988 | -0.117 | 0.516 |
|  | *Irs2* | -0.095 | 0.599 | -0.086 | 0.634 |
|  | *Isl1* | -0.128 | 0.476 | -0.259 | 0.145 |
|  | *Jak2* | 0.271 | 0.127 | 0.132 | 0.463 |
|  | *Jun* | -0.532 | 0.001 | 0.090 | 0.618 |
|  | *Junb* | 0.195 | 0.276 | -0.142 | 0.431 |
|  | *Jund* | -0.332 | 0.059 | -0.036 | 0.842 |
|  | *Kat2b* | 0.421 | 0.015 | 0.008 | 0.963 |
|  | *Kcnb1* | -0.028 | 0.877 | 0.024 | 0.894 |
|  | *Kit* | -0.201 | 0.263 | -0.151 | 0.401 |
|  | *Lepr* | -0.026 | 0.884 | 0.104 | 0.563 |
|  | *Mapk8* | -0.126 | 0.483 | 0.134 | 0.456 |
|  | *Mc3r* | -0.298 | 0.092 | -0.141 | 0.435 |
|  | *Mc4r* | -0.068 | 0.707 | -0.096 | 0.596 |
|  | *Mgrn1* | -0.163 | 0.365 | -0.186 | 0.300 |
|  | *Mmp9* | -0.298 | 0.092 | -0.072 | 0.691 |
|  | *Mrap2* | -0.242 | 0.175 | -0.066 | 0.713 |
|  | *Msx1* | 0.437 | 0.011 | -0.076 | 0.674 |
|  | *Mtor* | -0.286 | 0.106 | -0.093 | 0.607 |
|  | *Ncoa2* | -0.341 | 0.052 | 0.103 | 0.568 |
|  | *Ncoa3* | -0.130 | 0.469 | 0.238 | 0.182 |
|  | *Ncor1* | -0.272 | 0.125 | 0.035 | 0.848 |
|  | *Ncor2* | -0.172 | 0.338 | -0.054 | 0.764 |
|  | *Negr1* | -0.274 | 0.122 | -0.058 | 0.750 |
|  | *Neurod1* | 0.197 | 0.273 | <0.001 | 0.998 |
|  | *Npbwr1* | -0.402 | 0.020 | 0.056 | 0.757 |
|  | *Npm1* | 0.361 | 0.039 | -0.009 | 0.962 |
|  | *Npy1r* | 0.194 | 0.279 | 0.060 | 0.740 |
|  | *Npy2r* | -0.297 | 0.093 | -0.275 | 0.121 |
|  | *Npy5r* | 0.092 | 0.611 | -0.235 | 0.187 |
|  | *Nr2f1* | 0.028 | 0.877 | -0.175 | 0.329 |
|  | *Nr3c1* | -0.255 | 0.151 | -0.052 | 0.774 |
|  | *Nr4a1* | 0.368 | 0.035 | -0.088 | 0.626 |
|  | *Nr4a2* | -0.248 | 0.164 | -0.037 | 0.836 |
|  | *Nr5a1* | -0.119 | 0.511 | 0.109 | 0.546 |
|  | *Ogfr* | 0.210 | 0.240 | -0.208 | 0.245 |
|  | *Oprd1* | -0.205 | 0.252 | 0.129 | 0.474 |
|  | *Oprk1* | -0.202 | 0.259 | -0.123 | 0.496 |
|  | *Oprm1* | -0.396 | 0.022 | 0.210 | 0.240 |
|  | *Otx2* | 0.389 | 0.025 | -0.132 | 0.462 |
|  | *Oxt* | -0.180 | 0.315 | -0.163 | 0.365 |
|  | *Oxtr* | -0.305 | 0.085 | -0.062 | 0.733 |
|  | *Pcsk1* | -0.333 | 0.059 | 0.078 | 0.667 |
|  | *Pcsk2* | 0.048 | 0.789 | -0.468 | 0.006 |
|  | *Penk* | 0.151 | 0.401 | -0.087 | 0.631 |
|  | *Pgr* | -0.424 | 0.014 | 0.127 | 0.483 |
|  | *Pick1* | -0.413 | 0.017 | -0.098 | 0.589 |
|  | *Pik3r1* | 0.123 | 0.495 | 0.045 | 0.802 |
|  | *Pitx2* | -0.214 | 0.232 | -0.145 | 0.422 |
|  | *Pmch* | -0.084 | 0.643 | -0.094 | 0.604 |
|  | *Pml* | -0.182 | 0.311 | 0.188 | 0.296 |
|  | *Pou4f1* | -0.075 | 0.679 | 0.065 | 0.719 |
|  | *Ppargc1a* | 0.024 | 0.893 | -0.157 | 0.384 |
|  | *Prkcd* | 0.116 | 0.520 | -0.306 | 0.083 |
|  | *Pten* | -0.247 | 0.165 | 0.213 | 0.233 |
|  | *Ptk2b* | 0.202 | 0.260 | -0.060 | 0.740 |
|  | *Ptpn11* | 0.200 | 0.265 | 0.027 | 0.883 |
|  | *Ptpn6* | -0.069 | 0.703 | -0.010 | 0.958 |
|  | *Rara* | -0.085 | 0.639 | -0.057 | 0.754 |
|  | *Rarb* | 0.020 | 0.912 | -0.096 | 0.593 |
|  | *Rb1* | -0.040 | 0.825 | 0.150 | 0.406 |
|  | *Rcor1* | -0.237 | 0.185 | 0.122 | 0.499 |
|  | *Rest* | -0.238 | 0.183 | 0.262 | 0.140 |
|  | *Runx2* | 0.056 | 0.759 | 0.168 | 0.350 |
|  | *Rxra* | -0.019 | 0.917 | -0.023 | 0.901 |
|  | *Scg5* | 0.336 | 0.056 | -0.340 | 0.053 |
|  | *Sdc1* | -0.048 | 0.791 | -0.013 | 0.945 |
|  | *Sh2b1* | -0.353 | 0.044 | -0.227 | 0.204 |
|  | *Sim1* | -0.272 | 0.126 | 0.067 | 0.710 |
|  | *Sin3a* | -0.202 | 0.260 | -0.228 | 0.202 |
|  | *Sirt1* | 0.305 | 0.085 | -0.025 | 0.892 |
|  | *Slc18a2* | -0.291 | 0.100 | -0.080 | 0.658 |
|  | *Slc6a3* | -0.368 | 0.035 | -0.071 | 0.694 |
|  | *Smad3* | 0.114 | 0.527 | 0.139 | 0.440 |
|  | *Smarca4* | -0.501 | 0.003 | -0.071 | 0.694 |
|  | *Snta1* | -0.040 | 0.824 | -0.103 | 0.568 |
|  | *Socs3* | -0.005 | 0.978 | 0.087 | 0.632 |
|  | *Sp1* | -0.347 | 0.048 | 0.209 | 0.242 |
|  | *Sst* | 0.018 | 0.921 | -0.167 | 0.353 |
|  | *Sstr2* | -0.058 | 0.749 | 0.071 | 0.693 |
|  | *Stat1* | -0.615 | <0.001 | 0.115 | 0.524 |
|  | *Stat3* | -0.155 | 0.389 | -0.029 | 0.872 |
|  | *Stat5b* | -0.479 | 0.005 | -0.186 | 0.300 |
|  | *Tac1* | 0.228 | 0.203 | 0.036 | 0.843 |
|  | *Tcf3* | -0.204 | 0.254 | 0.188 | 0.294 |
|  | *Th* | -0.378 | 0.030 | -0.056 | 0.756 |
|  | *Thra* | 0.304 | 0.085 | 0.011 | 0.953 |
|  | *Thrb* | -0.238 | 0.182 | 0.043 | 0.814 |
|  | *Tmem18* | 0.300 | 0.089 | 0.114 | 0.529 |
|  | *Traf4* | -0.379 | 0.030 | -0.059 | 0.743 |
|  | *Trh* | -0.344 | 0.050 | -0.079 | 0.660 |
|  | *Trhr* | -0.507 | 0.003 | 0.081 | 0.654 |
|  | *Tsc22d3* | 0.454 | 0.008 | 0.140 | 0.438 |
|  | *Ubc* | -0.371 | 0.034 | -0.135 | 0.454 |
|  | *Ucp2* | 0.163 | 0.364 | 0.085 | 0.636 |
|  | *Vgf* | -0.050 | 0.780 | -0.239 | 0.180 |
|  | *Zbtb16* | 0.400 | 0.021 | 0.081 | 0.654 |
